# Modified creatinine index for predicting prognosis in hemodialysis patients: a systematic review and meta-analysis

**DOI:** 10.1080/0886022X.2024.2367026

**Published:** 2024-08-09

**Authors:** Tao Ye, Jingfang Du, Pian Li, Dan Rong, Wang Gu, Yao Yao, Na Shen

**Affiliations:** a School of Clinical Medicine, Hebei University of Engineering, Handan, China; b Emergency Department of Wangcang County People’s Hospital, Guangyuan City, China; c Affiliated Hospital of Hebei Engineering University, Handan, China

**Keywords:** Modified creatinine index, hemodialysis, prognosis, meta-analysis

## Abstract

**Background:**

Currently, several studies have explored the association between the modified creatinine index (mCI) and prognosis in patients on hemodialysis (HD). However, some of their results are contradictory. Therefore, this study was conducted to comprehensively assess the role of mCI in predicting prognosis in HD patients through meta-analysis.

**Methods:**

We searched and screened literature from PubMed, Embase, Web of Science, and Cochrane databases from their establishment until March 2024. Relevant data were extracted. The statistical analysis was performed using Stata 15.0, RevMan 5.4, and Meta DiSc 1.4 software.

**Results:**

The results showed a positive association between mCI and nutritional status in HD patients (BMI *r* = 0.19, 95% CI: 0.1–0.28, *p* = .000; albumin *r* = 0.36, 95% CI: 0.33–0.39, *p* = .000; normalized protein catabolic rate (nPCR) *r* = 0.25, 95% CI: 0.13–0.38, *p* = .000). In addition, mCI in deceased HD patients was significantly lower than that in HD survivors (SMD = −0.94, 95% CI: −1.46 to −0.42, *p* = .000). A low mCI was associated with an increased risk of all-cause death in HD patients (HR = 1.95, 95% CI: 1.57–2.42, *p* = .000). In addition, a low mCI was significantly associated with decreased overall survival (OS) in HD patients (HR = 3.01, 95% CI: 2.44–3.70, *p* = .000). mCI showed moderate diagnostic accuracy for sarcopenia in both male and female HD patients (male AUC = 0.7891; female AUC = 0.759).

**Conclusions:**

The mCI can be used as a prognostic marker for HD patients, and monitoring mCI may help to optimize the management of HD and improve overall prognosis in patients.

## 1. Introduction

Hemodialysis (HD), a form of renal replacement therapy (RRT), is currently the main choice for patients with end-stage renal disease (ESRD). Currently, nearly 4 million people in the world are undergoing RRT, and HD patients account for 69% of them [1]. Most ESRD patients still require long-term and even lifelong HD, although it relieves their disease. During HD, patients often present with symptoms of malnutrition including loss of body mass, muscle loss, and protein energy wasting (PEW) [2], which are accompanied by a higher risk of infection/death and a lower survival rate, as compared with those in the normal population [3]. In addition, with the increasing trend of global aging, the incidence of ESRD has risen, which in turn has led to a change in the age distribution of HD patients [4]. In Japan, one of countries with the most severely aging population, the mean age of HD patients reached 69 years as early as 2017 [5]. This undoubtedly makes it more difficult to manage HD patients. Given these serious prognostic and societal burdens, there is a need to identify a valid and accurate indicator to screen HD patients for adverse clinical events.

In 1995, Canaud et al. obtained a normalized creatinine production rate, the creatinine index (CI), which is equal to the sum of the estimated creatinine excretion rate and the metabolic degradation rate and was derived from creatinine kinetic modeling (CKM) and used to assess nutritional status in HD patients [6]. Subsequently, in a study by Desmeules et al., CI became a predictor of long-term survival and mortality in HD patients [7]. However, its cumbersome calculation formula has become a major problem for clinical use. Until 2014, Canaud et al. succeeded in simplifying the CI formula (modified CI, termed as mCI) simply by using some usual data, i.e., age, gender, pre-dialysis serum creatinine level, and urea clearance index (*Kt*/*V*) [7]. Consequently, in the following 10 years or so, mCI were frequently used in clinical studies in HD patients, but whether it is associated with prognosis in HD patients is still controversial. For example, a study by Bataille et al. stated that mCI could not predict sarcopenia in HD patients [8], whereas studies by Tian et al. and Kakita et al. stated that mCI could be used to predict sarcopenia in HD patients [9,10].

Therefore, we summarized currently available literature relevant to the topic and initially performed a systematic review and meta-analysis of it to assess the association between mCI and prognosis in HD patients.

## 2. Methods

### 2.1. Search strategy

This study was already registered on PROSPERO (CRD42024529310) and strictly followed the Preferred Reporting Items for Systematic Reviews and Meta-analyses (PRISMA) reporting guideline. Using the same search strategy, two investigators conducted independent comprehensive search in Web of Science, Embase, Cochrane Library, and PubMed from inception to March 2024. Search terms included the subject headings ‘Renal Dialysis’ and ‘Creatinine’ and the free-text terms ‘haemodialysis, hemodialysis, Kreatinine, Krebiozen’, as shown in Attachment 1. Subsequently, no other publications were found after reference lists and citations were searched. Independent comprehensive search was conducted in Web of Science, Embase, Cochrane Library, PubMed, and Scopus from inception to March 2024. Search terms included the subject headings ‘Renal Dialysis’ and ‘Creatinine’ and the free-text terms ‘haemodialysis, hemodialysis, Kreatinine, Krebiozen’, as shown in Attachment 1. Subsequently, no other publications were found after reference lists and citations were searched.

### 2.2. Inclusion and exclusion criteria

Inclusion criteria: (1) study type: a cohort study; (2) study population: (2) adults (≥18 years) treated with HD; (3) formula for calculation of mCI: modified Cr index (mg/kg/day) = 16.21 + 1.12 × [1 if male; 0 if female] − 0.06 × age (years) − 0.08 × single-pooled *Kt*/*V* for urea + 0.009 × serum Cr level before dialysis (µmol/L); (4) studies evaluating the association between mCI and prognosis in HD patients. Exclusion criteria: (1) reviews, letters, pathology reports, conference abstracts, meta-analyses, or animal studies; (2) studies conducted by the same authors using the same data; (3) unavailable full texts or insufficient data.

### 2.3. Literature screening

In this study, duplicate references were first removed, and then, the titles and abstracts of literature were independently read by two investigators for initial screening based on pre-established inclusion and exclusion criteria. Finally, the full texts were read to exclude nonconforming literature, thereby identifying final literature to be included and extracting relevant data.

### 2.4. Data extraction

Data extraction in this study was performed independently by two investigators. The main data extracted included the first author, year of publication, region where a study was conducted, specific type of a study, sample size, mean age, sex ratio, and relevant outcome measures, such as risk of all-cause death, and sensitivity and specificity to predict sarcopenia. If disagreements arose between the two investigators (Tao Ye and Jingfang Du) during this process, they would be resolved by a third investigator (Pian Li).

### 2.5. Quality assessment

In this study, two investigators (Tao Ye and Jingfang Du) independently assessed the risk of bias in included studies using the Newcastle Ottawa Scale (NOS) and Joanna Briggs Institute Practical Application of Clinical Evidence System (JBI PACES) [11]. NOS assessment contains the following: adequacy of the case definition, representativeness of the cases, selection of controls, comparability of cases and controls, and ascertainment of exposure. In addition, based on the corresponding scores, the included studies were classified as high quality (7–9), medium quality (4–6), and low quality (1–3). JBI assessment contains 10 items: ① Was the purpose of the study clear? Was the study fully justified? ② How was the study population selected? ③ Were the inclusion and exclusion criteria for samples clearly described? ④ Were the sample characteristics clearly described? ⑤ Were the data collection instruments reliable and valid? ⑥ What were the measures to verify the authenticity of the data? ⑦ Were ethical issues taken into account? ⑧ Were the statistical methods correct? ⑨ Were the results of the study presented appropriately and accurately? ⑩ Was the value of the study clearly stated? Each item was assessed by ‘non-compliant (0),’ ‘mentioned, but not described in detail (1)’, and ‘described fully and accurately in detail (2)’. When the score was > 70% of the total score, i.e., a score >14, it was considered that the study had a low risk of bias. If disagreements arose between the two investigators, they would be resolved via negotiation with a third investigator (Ping Li).

### 2.6. Statistical analysis

All statistical analyses were performed using Stata 15.0 (StataCorp, College Station, TX), RevMan 5.4 (The Cochrane Centre Collaboration, Copenhagen, Denmark), and Meta-DiSc 1.4 (Madrid, Spain). Multivariable-adjusted effect estimates (odds ratio (OR)/hazard ratio (HR)) were extracted for all included studies to calculate the pooled estimates and 95% confidence intervals (CIs). In addition, the standardized mean difference (95% CI) of mCI between groups such as the surviving group and the dead group was assessed. The *I*^2^ statistic was used to measure the level of heterogeneity. *I*^2^ > 50% indicated significant heterogeneity among the included studies. For it, a random-effects model was used, and sensitivity analysis (one-by-one elimination method), meta-regression (*n* ≥ 5) and subgroup analysis (*n* ≥ 2) were performed to find the source of heterogeneity. If *I*^2^ < 50%, a fixed-effects model would be used. Publication bias in included studies was assessed by a funnel plot (*n* > 8), Egger’s test and Begg’s test. Egger’s or Begg’s *p* < .05 was considered to indicate the presence of publication bias. If publication bias was present, publication bias would be corrected by the trim-and-fill method.

## 3. Results

### 3.1. Literature search results

**Figure 1.**
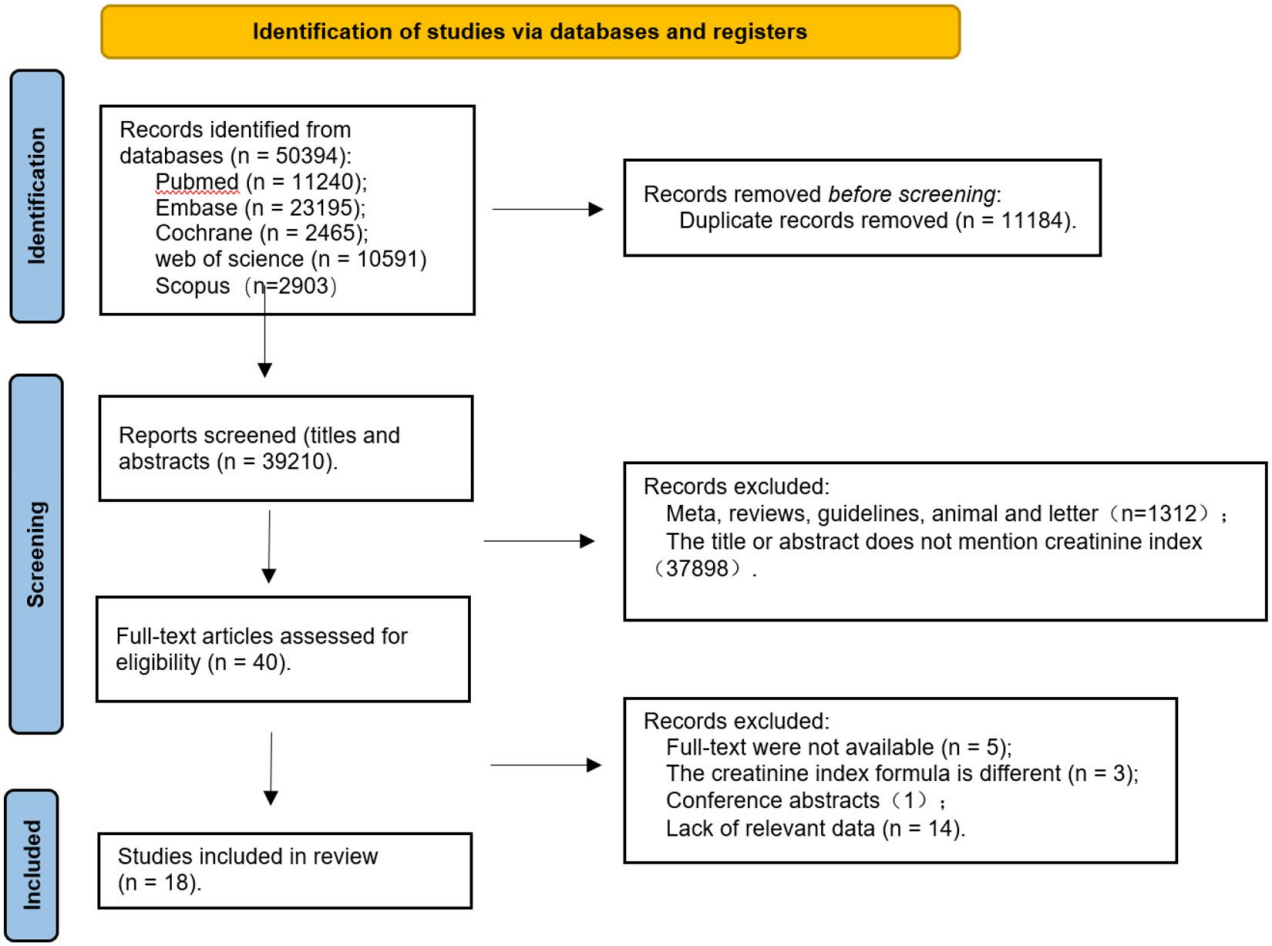
Literature screening process.

According to the search strategy, a total of 50,394 references were obtained. Of them, 11,240 were published in PubMed, 23,195 published in EMBASE, 2465 published in Cochrane, 10,591 published in Web of Science, 2903 published in Scopus, and 11,184 duplicates removed. Forty references were obtained after the titles and abstracts were read. Their quality was then assessed based on their full texts. Twenty-two of them were excluded, including five references without full texts available, three references containing different CI formulas, one conference abstract, and 13 references without relevant data. In the end, 18 references [8–10,12–26] were obtained. The literature screening process is shown in Figure 1.

### 3.2. Characteristics of the included studies

Of the 80 references (studies) included, 15 were published in Asia, two were published in Europe, and one was published in Oceania. They were published from 2016 to 2023. They involved 32,160 patients. They were all cohort studies. The included studies were given NOS scores and JBI scores separately. NOS scores showed 15 high-quality studies [8–10,12,14–24,26] and three medium-quality studies [13,15,25]. JBI scores showed all the included studies were scored >14, with a low risk of bias. Data are detailed in Table 1.

**Table 1.** General characteristics of included studies.!!

Included reference (study)	Region	Study type	Total sample size (males/females, *n* (*n*/*n*))	Age (mean ± SD)	mCI (mg/kg/day) cutoff value	NOS score	JBM score	Outcome measure
Arase et al. [11]	Japan	Cohort study	3027 (1793/1234)	64.32 ± 12.52	–	7	15	①②③
Bataille et al. [8]	France	Cross-sectional study	111 (65/46)	77.71 ± 10.54	–	8	19	⑨⑩
Canaud et al. [16]	European	Retrospective cohort study	23,495 (13,392/10,103)	61 ± 15	20.5	6	15	⑤
Chandler et al. [12]	Australia	Retrospective study	179 (99/80)	60.06 ± 14.95	18.55	8	17	①②④
Ebinç et al. [17]	Turkey	Retrospective, cross-sectional study	169 (85/84)	57.09 ± 16.19	12.18	6	16	⑤⑦
Fujioka et al. [19]	Japan	Retrospective study	183 (98/85)	68.3 ± 12.4	21.1	7	15	⑥⑧
Fukushi et al. [18]	Japan	Retrospective study	284 (198/95)	68.3 ± 11.92	20.6	7	15	⑤⑥⑧
Huang et al. [13]	China	Retrospective cohort study	1269 (642/627)	61.67 ± 11	–	8	19	①②③
Hwang et al. [14]	South Korea	Retrospective study	88 (45/44)	56.9 ± 16.6	–	7	16	③
Kakita et al. [9]	Japan	Cross-sectional study	356 (227/129)	70.49 ± 12.13	20.05	8	15	④⑨⑩
Naito et al. [20]	Japan	Retrospective, observational study	499 (334/165)	65 ± 13.38	21	8	19	⑥⑧
Suzuki et al. [21]	Japan	Retrospective cohort study	472 (297/175)	63.8 ± 13.7	20.3	8	18	④⑥⑦
Suzuki et al. [22]	Japan	Retrospective cohort study	349 (213/136)	67.4 ± 13.1	21.2	7	18	④⑥⑦
Tian et al. [10]	China	Cross-sectional study	346 (213/133)	58.17 ± 13.77	20.32	9	17	⑤⑧⑨⑩
Tsai et al. [15]	China	Cross-sectional study	230 (150/80)	64.0 ± 14.3	–	7	19	①
Yajima and Yajima [23]	Japan	Retrospective study	224 (149/75)	63.3 ± 13.9	20.4	8	18	⑥
Yajima et al. [24]	Japan	Retrospective cohort study	263 (175/88)	63.8 ± 13.7	20.16	6	16	⑥⑦
Yamamoto et al. [25]	Japan	Retrospective study	542 (327/215)	65.3 ± 12.1	20.08	8	16	④⑥⑦

① Association between mCI and albumin; ② association between mCI and BMI; ③ association between mCI and nPCR; ④ association between mCI and the duration of maintenance of dialysis in HD patients; ⑤ difference in the level of mCI between surviving and dead patients; ⑥ association between mCI and risk of all-cause death in patients; ⑦ association between mCI in the continuous model and risk of all-cause death in patients; ⑧ association between mCI and OS in patients; ⑨ sensitivity and specificity of mCI in predicting sarcopenia in male patients; ⑩ sensitivity and specificity of mCI in predicting sarcopenia in female patients.

### 3.3. Association between mCI and the duration of maintenance of dialysis in HD patients

**Figure 2.**
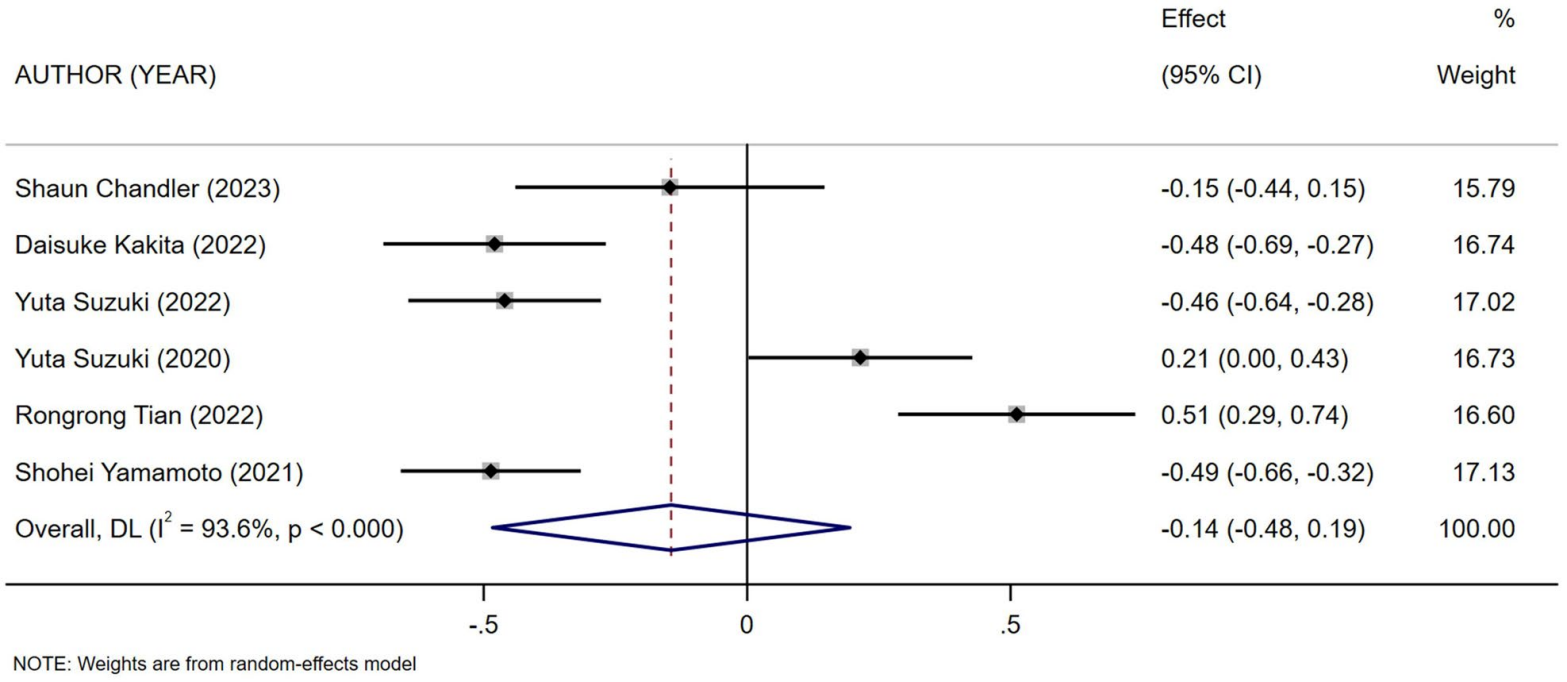
Forest plot for the association between low and high levels of mCI and the duration of maintenance of dialysis in HD patients.

Six studies [9,10,14,21,22,26] described the duration of maintenance of dialysis in HD patients in high and low mCI groups. The heterogeneity test showed high heterogeneity (*I*^2^ = 93.6%). Therefore the random-effects model was used for meta-analysis. The results showed that there was no association between high and low levels of mCI and the duration of maintenance of dialysis in HD patients (SMD = −0.14, 95% CI: −0.48 to 0.19, *p* = .404), as shown in Figure 2. Sources of heterogeneity were subsequently explored based on sensitivity analysis (one-by-one elimination method), meta-regression (mean age of the study population, mCI cutoff value), and subgroup analysis (mean age of the study population, type of the study). No sources of heterogeneity sensitivity were shown by sensitivity and subgroup analyses (Attachment 1), and meta-regression (mean age, *p* = .275; mCI cutoff value, *p* = .803). Tests for publication bias were performed. The results showed no publication bias (Egger’s test, *p* = .323; Begg’s test, *p* = .06).

### 3.4. Association between mCI and nutritional status in HD patients

**Figure 3.**
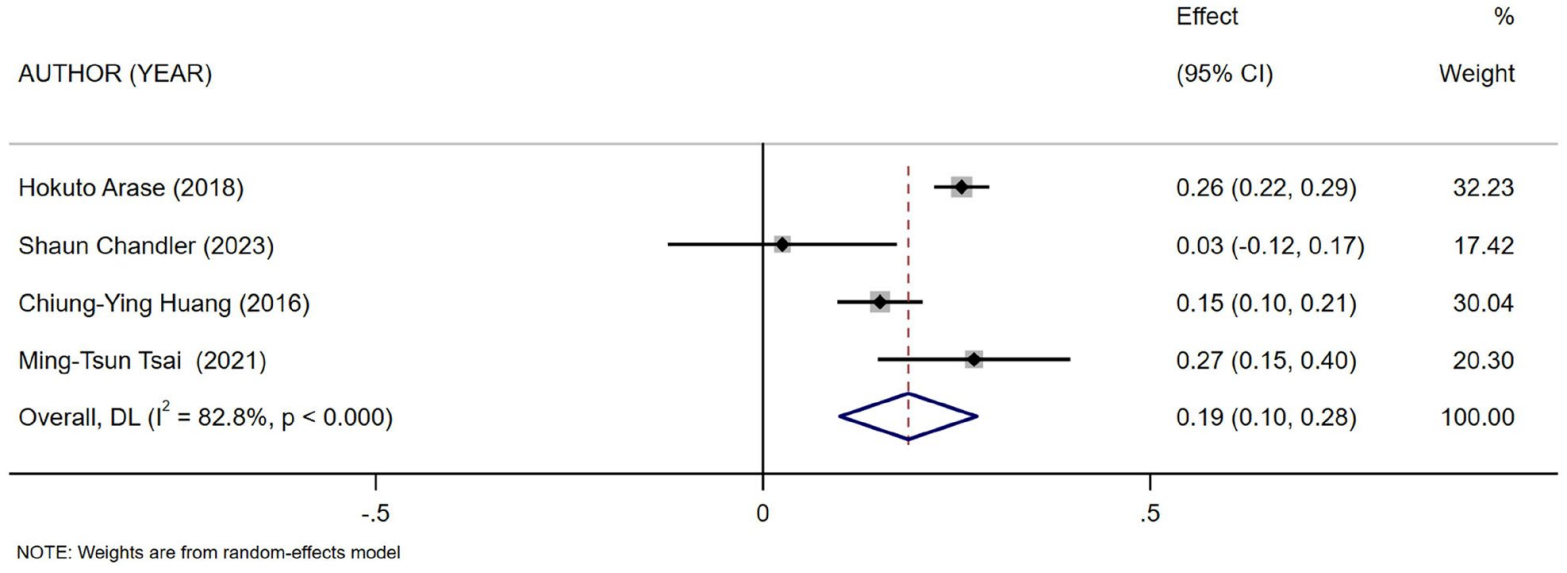
Forest plot for the association between mCI and BMI.

**Figure 4.**
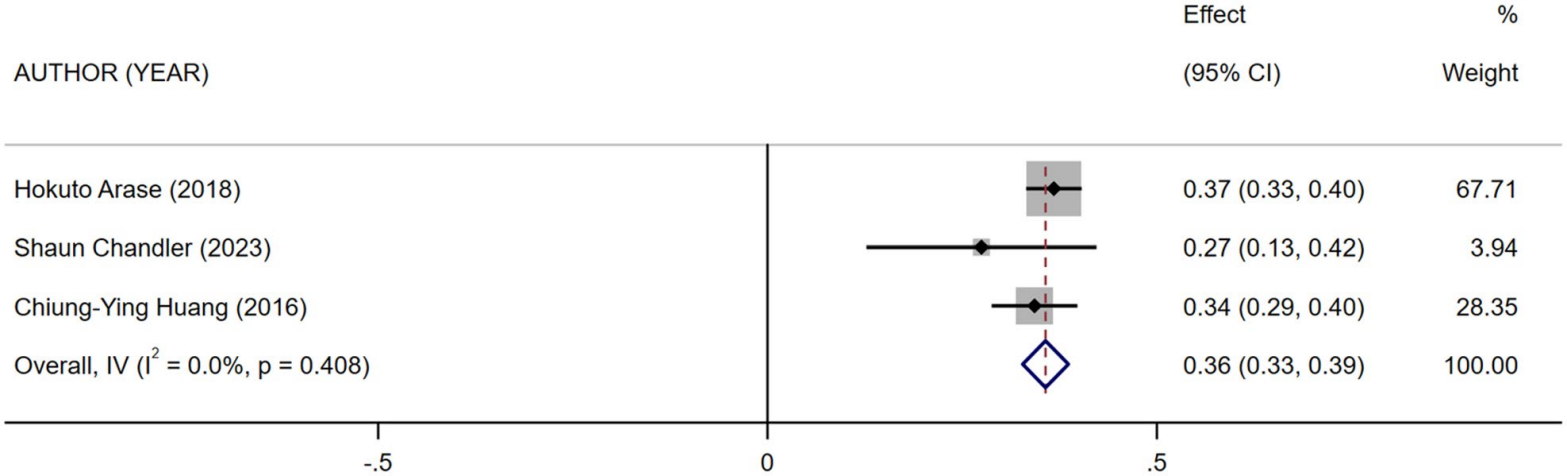
Forest plot for the association between mCI and albumin.

**Figure 5.**
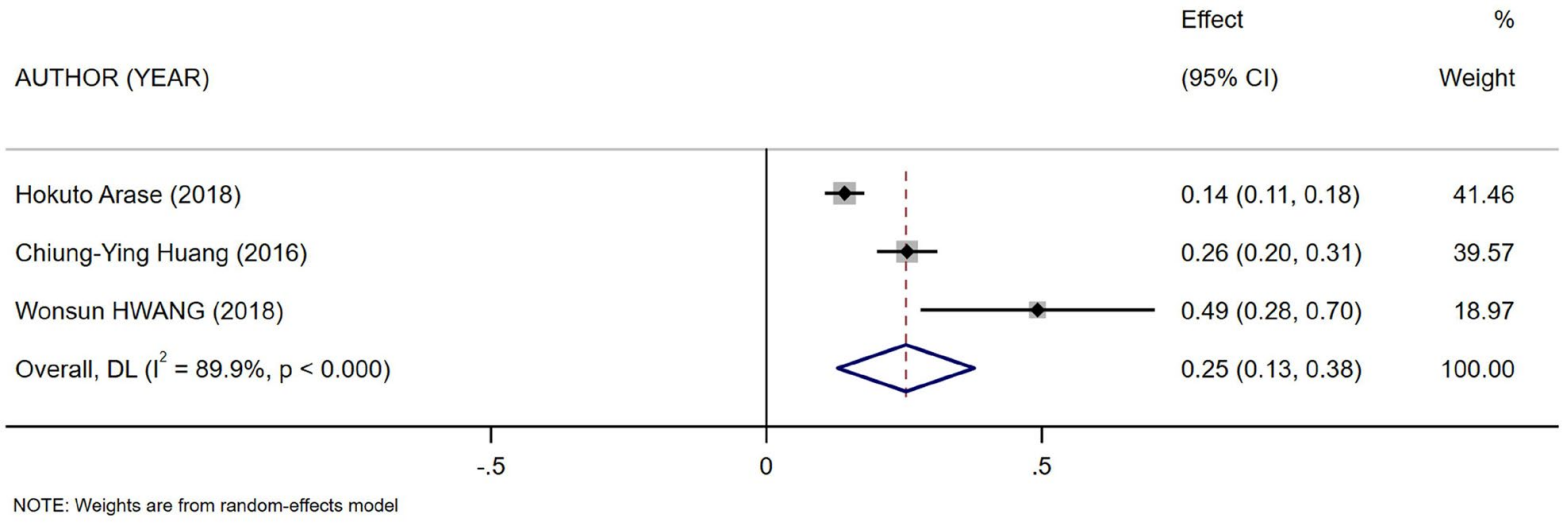
Forest plot for the association between mCI and nPCR.

Five references [11–15] described the association between mCI and indicators of nutritional status in HD patients: body mass index (BMI), albumin, and normalized protein catabolic rate (nPCR). Of them, four [11–13,15] reported the association between mCI and BMI (*I*^2^ = 82.8%), three [11,13,14] reported the association between mCI and nPCR (*I*^2^ = 89.9%), and three [11–13] reported the association between mCI and albumin (*I*^2^ = 0.0%). The tests for heterogeneity in BMI and nPCR were performed using the random-effects model. The test for heterogeneity in albumin was performed using the fixed-effects model. The results showed that mCI was positively associated with BMI (*r* = 0.19, 95% CI: 0.1–0.28, *p* = .000), albumin (*r* = 0.36, 95% CI: 0.33–0.39, *p* = .000), and nPCR (*r* = 0.25, 95% CI: 0.13–0.38, *p* = .000), as shown in Figures 3–5. Subsequently, sensitivity analysis and a test for publication bias were performed. No sources of heterogeneity were found (BMI: Egger’s test, *p* = .451, Begg’s test, *p* = .734; nPCR, Egger’s test, *p* = .324, Begg’s test, *p* = 1; albumin, Egger’s test, *p* = .122, Begg’s test, *p* = .296).

### 3.5. Difference in the level of mCI between surviving and dead HD patients

**Figure 6.**
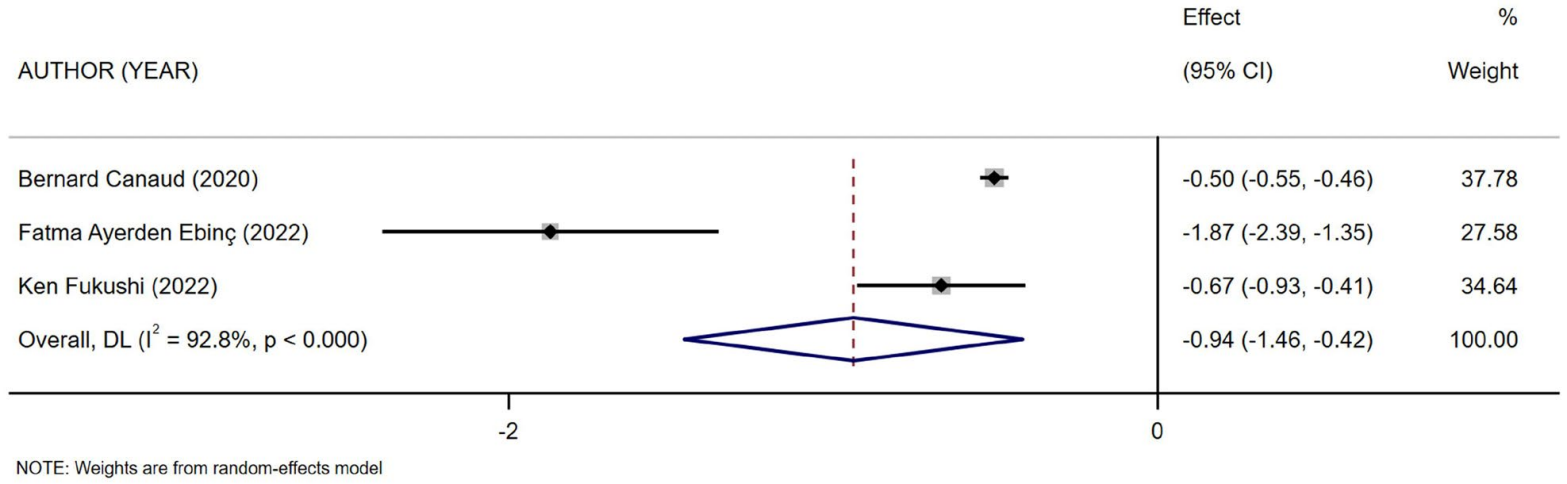
Forest plot for the association between mCI and survival/death in HD patients.

Three references [16–18] described the level of mCI in surviving and dead HD patients. The heterogeneity test showed high heterogeneity (*I*^2^ = 92.8%). Therefore, the random-effects model was used for analysis. The results showed a difference in the level of mCI between surviving and dead HD patients (SMD = −0.94, 95% CI: −1.46 to −0.42, *p* = .000), as shown in Figure 6. Subsequently, sensitivity analysis and a test for publication bias were performed. The results showed no sources of heterogeneity, and absence of publication bias (Egger’s test, *p* = .322, Begg’s test, *p* = .296).

### 3.6. Association between mCI and risk of all-cause death/OS in HD patients

**Figure 7.**
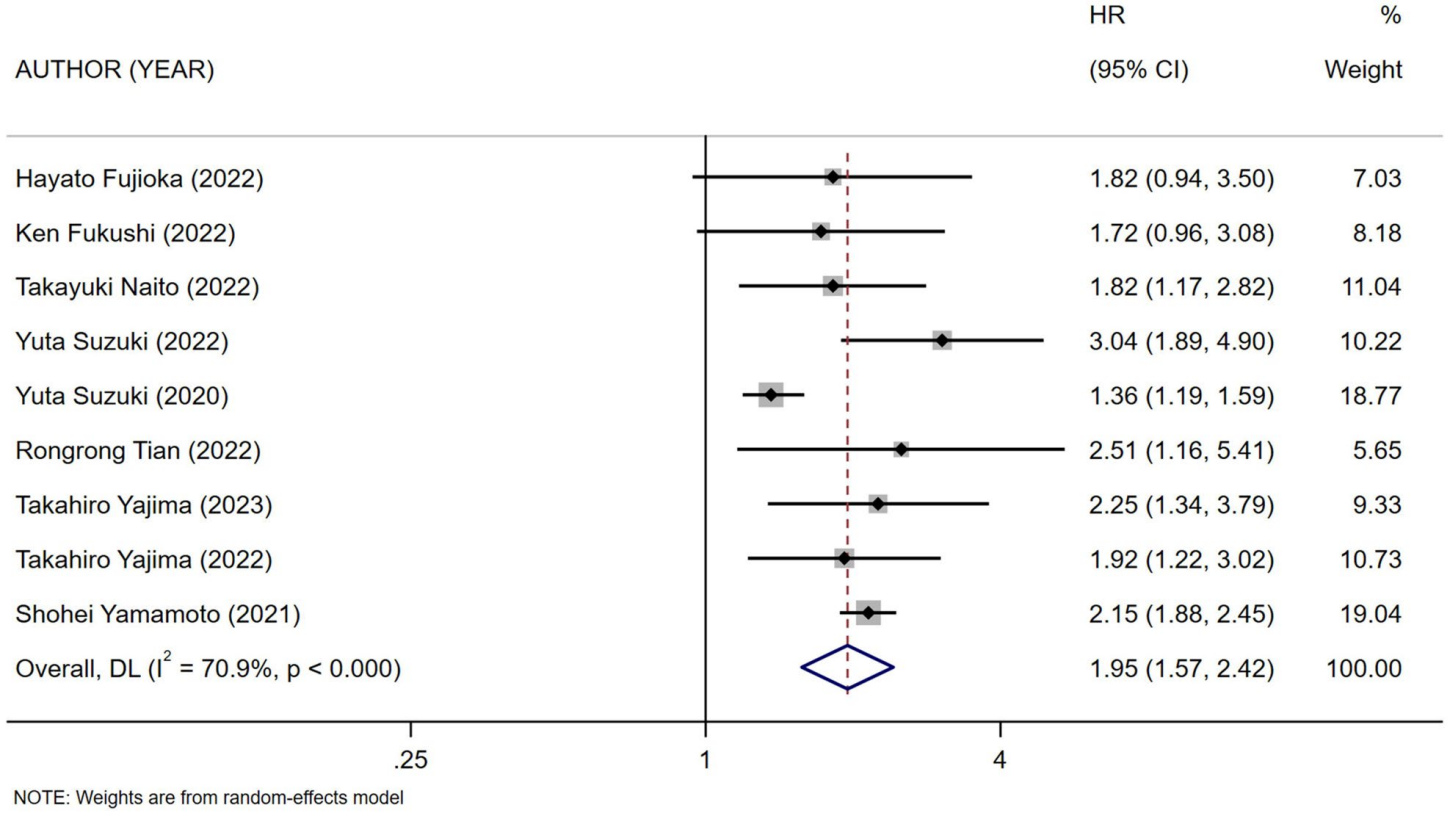
Forest plot for the association between mCI and risk of all-cause death in HD patients.

**Figure 8.**
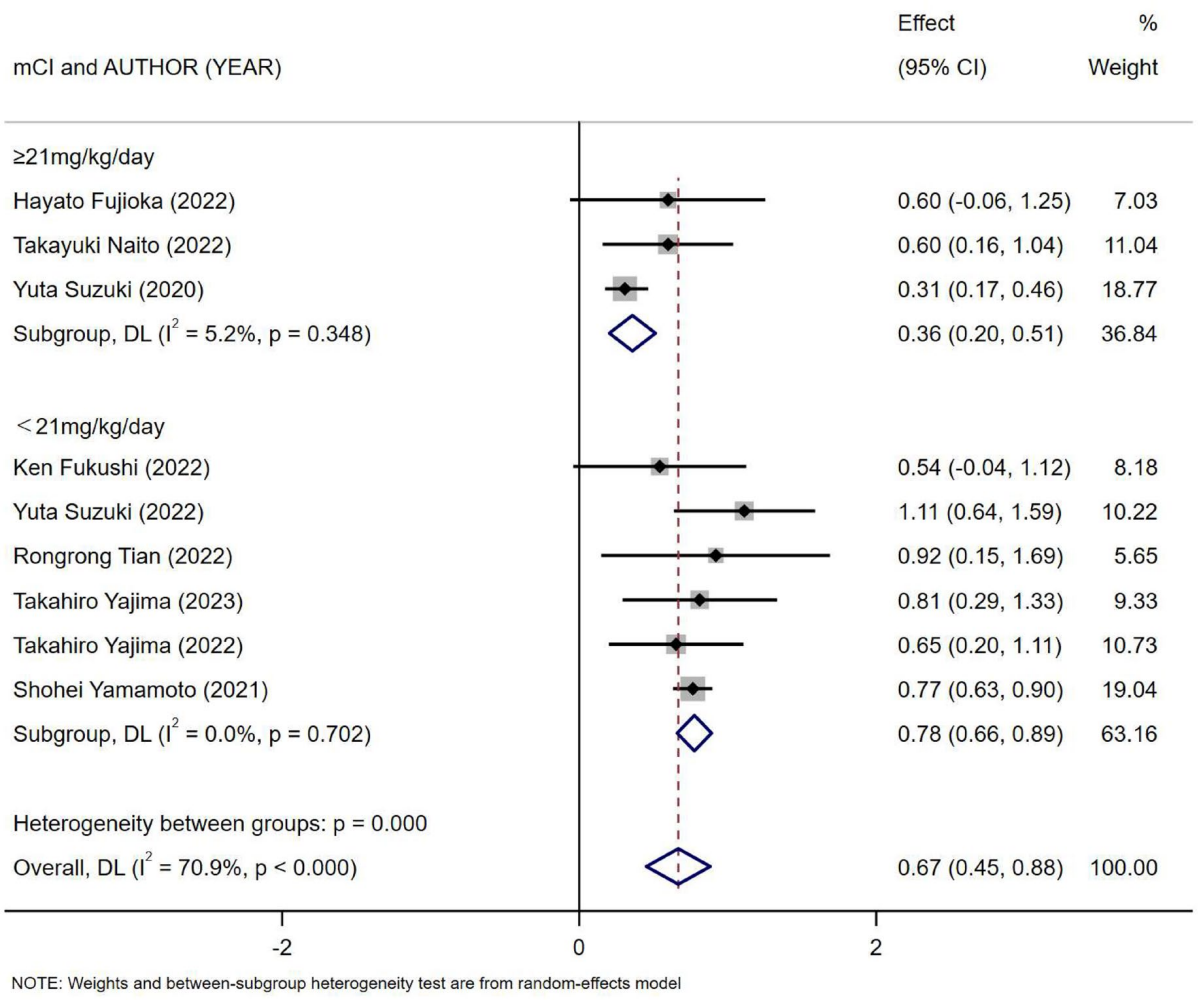
Forest plot for the association between mCI (21 mg/kg day) and risk of all-cause death in HD patients.

**Figure 9.**
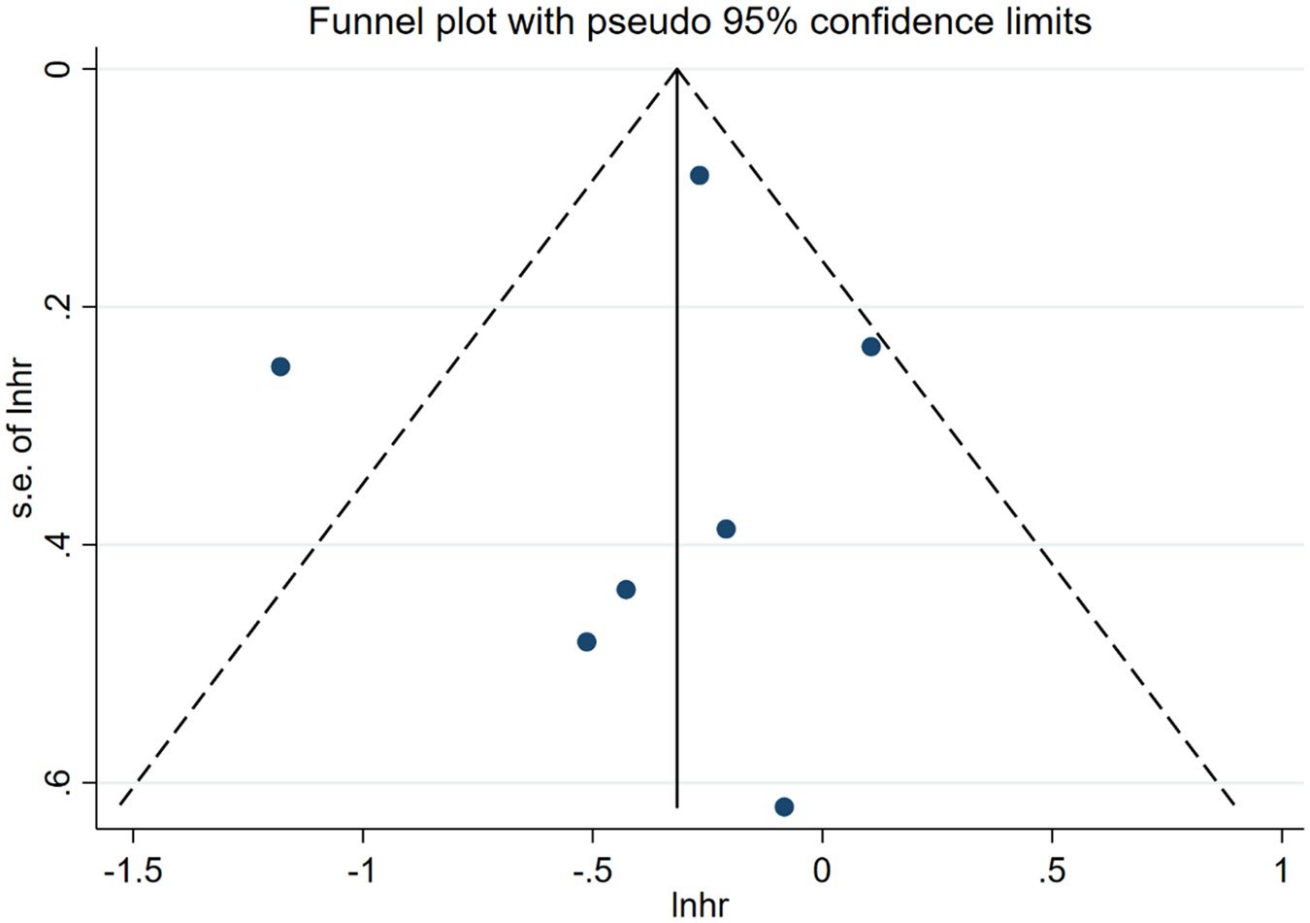
Test for publication bias in the association between mCI and risk of all-cause death in HD patients.

**Figure 10.**
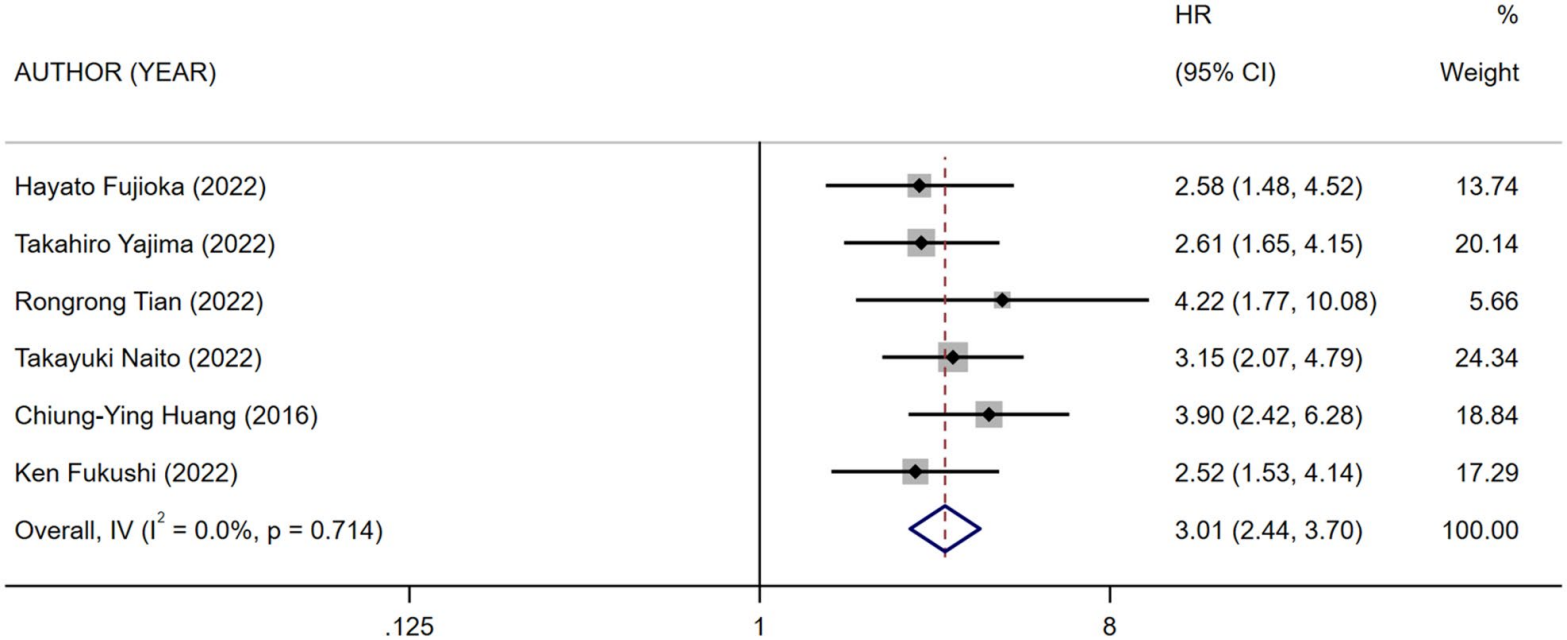
Forest plot for the association between mCI and OS in HD patients.

Nine references [10,18–25] described the association between mCI and risk of all-cause death in HD patients, and the heterogeneity test showed high heterogeneity in risk of all-cause death (*I*^2^ = 70.9%). Six references [10,13,18–20,24] described the association between mCI and overall survival (OS) in HD patients, and the heterogeneity test showed no heterogeneity in OS (*I*^2^ = 0%). The association between mCI and OS in HD patients was assessed using the fixed-effects model. The association between mCI and risk of all-cause death in HD patients was assessed using the random-effects model. The results showed that mCI was associated with risk of all-cause death (HR = 1.95, 95% CI: 1.57–2.42, *p* = .000) and OS (HR = 3.01, 95% CI: 2.44–3.70, *p* = .000) in HD patients. Subsequently, sensitivity analysis, meta-regression (mean age of the study population, mCI cutoff value) and subgroup analysis (mCI cutoff value) were performed regarding the risk of all-cause death in mCI and HD patients. As a result, sensitivity analysis and meta-regression showed that mCI was associated with the risk of all-cause mortality in HD patients (mean age of the study population, *p* = .065, mCI cutoff value, *p* = .004). Subgroup analysis (mCI cutoff value) showed mCI ≥21 mg/kg/day: *I*^2^ = 5.2%, HR = 0.36, 95% CI 0.20–0.51, *p* = .000; mCI <21 mg/kg/day: *I*^2^ = 0%, HR = 0.78, 95% CI 0.66–0.89, *p* = .000. Tests for publication bias showed the absence of publication bias (association between mCI and risk of all-cause death in HD patients: Egger’s test, *p* = .471, Begg’s test, *p* = 1; association between mCI and OS in HD patients: Egger’s test, *p* = .598, Begg’s test, *p* = 1), as shown in Figures 7–10.

### 3.7. Sensitivity and specificity of mCI in predicting sarcopenia in HD patients

**Figure 11.**
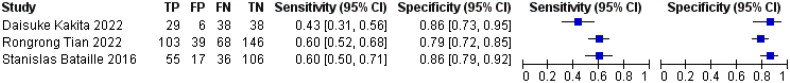
Sensitivity and specificity of mCI in predicting sarcopenia in male HD patients.

**Figure 12.**
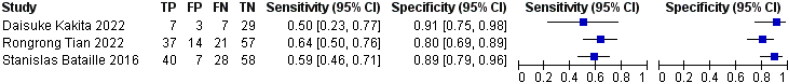
Sensitivity and specificity of mCI in predicting sarcopenia in female HD patients.

**Figure 13.**
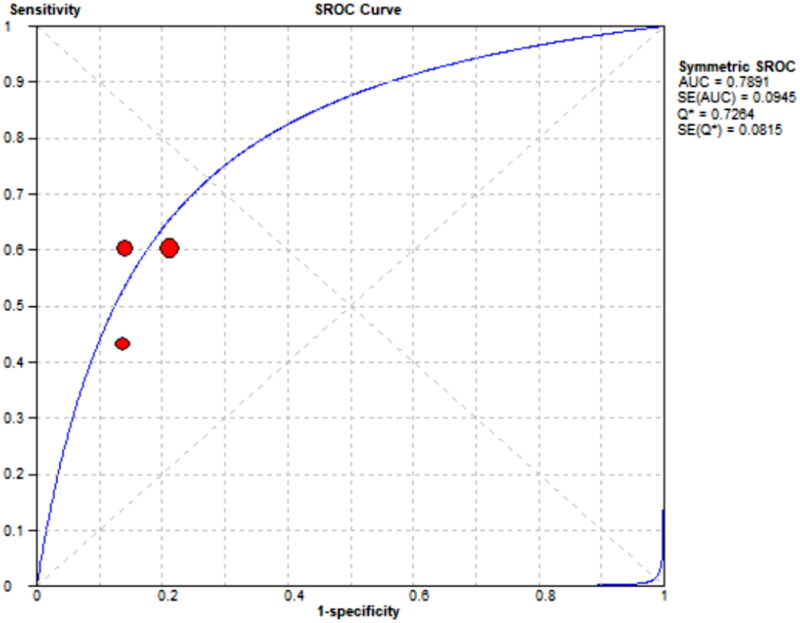
SROC curve for mCI in predicting sarcopenia in male HD patients.

**Figure 14.**
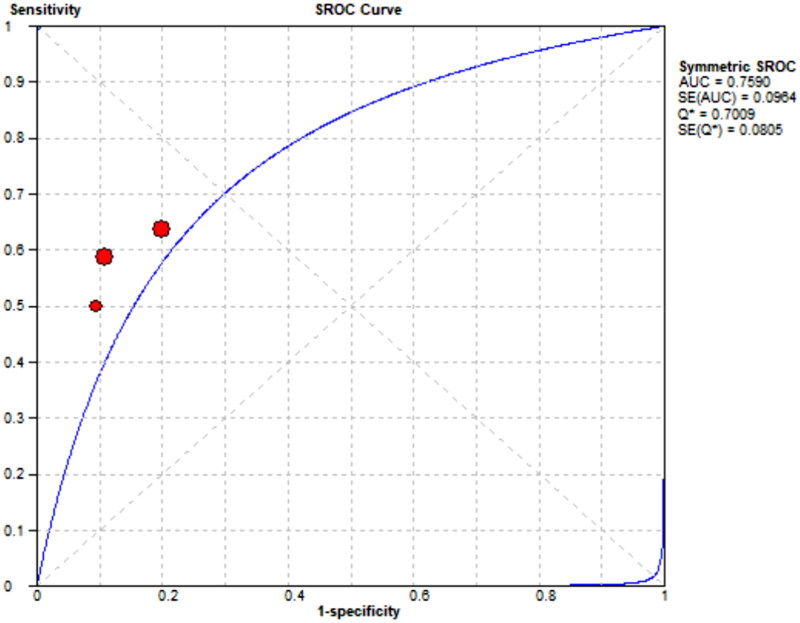
SROC curve for mCI in predicting sarcopenia in female HD patients.

Three references [8–10] respectively described the accuracy of mCI used to diagnose sarcopenia in HD patients. The results showed a sensitivity of 0.57 (95% CI: 0.51–0.62), specificity of 0.82 (95% CI: 0.78–0.86), and diagnostic odds ratio (DOR) of 6.43 (95% CI: 4.51–9.19; SROC curve AUC = 0.7891) for mCI in predicting sarcopenia in male HD patients, as well as a sensitivity of 0.60 (95% CI: 0.51–0.68), specificity of 0.86 (95% CI: 0.79–0.91), and DOR of 8.97 (95% CI: 5.12–15.74; SROC curve AUC = 0.759) for mCI in predicting sarcopenia in female HD patients, as shown in Figures 11–14.

## 4. Discussion

### 4.1. Nutritional status in HD patients

For half a century, HD has remained the mainstay of life-sustaining treatment (LST) for ESRD patients, but it is not a complete substitute for renal function. With long-term treatment with HD, such patients will have to face a serious problem with a prevalence of 28–54% – malnutrition [26]. Low-quality nutritional status may cause metabolic acidosis, altered intestinal microbial flora, chronic inflammation, accelerated degradation of muscle proteins, hormonal imbalance, and other alterations [27]. This not only impairs muscle mass but also increases the risk of infection and hospitalization in HD patients, thereby causing poor prognosis such as a decreased survival rate, increased risk of death, and occurrence of sarcopenia [28,29]. Currently, nutritional status in HD patients is often assessed clinically by parameters such as BMI, nPCR, and serum albumin [30]. In particular, the decreased level of serum albumin has been generally believed to be strongly associated with poor prognosis and higher risk of death in HD patients [31]. Our results showed that mCI was positively associated with BMI, nPCR, and serum albumin in HD patients. Therefore, it can be used for assessment of nutritional status in HD patients.

### 4.2. All-cause death and survival in HD patients

Currently, HD has improved dramatically, both technically and in terms of care management, but HD patients still face a high risk of death. In Poland only, HD patients had a mortality of up to 15–20%, which was 4–7 times that in the general population [32]. In contrast, the mortality was up to 25% within the first year of treatment with HD [33]. It is particularly significant in the first 90 days, with a staggeringly high percentage of 32–44% [34,35]. Meanwhile, the age-standardized incidence of ESRD treated with HD has increased by 43% with the global aging trend [36]. Statistics showed that patients older than 65 years of age accounted for 67.7% of dialysis patients in Portugal, which has the highest prevalence and incidence of ESRD in Europe [37]. Compared with young HD patients, older HD patients have a higher burden involving comorbidities, malnutrition and the efficiency of fistulae, thereby posing a higher risk of death and lower survival rate to older HD patients [1]. Therefore, there is an urgent need for an accurate parameter for predicting the probability of an outcome and for understanding how patients’ condition changes over time, so as to intervene in and improve the outcome in HD patients.

mCI is a parameter derived by Canaud et al. using the simplified CI formula. In a study by Huang et al., mCI was first proposed as a good parameter for predicting the survival rate in HD patients, and the level of mCI was found to be associated with the risk of death in HD patients [13]. Similar conclusions were also drawn from several subsequent cohort studies in Japan [19,20,23]. We pooled all currently available studies for meta-analysis. The results showed that mCI predicted all-cause death in HD patients. Therefore, it can be a potential predictor of survival in HD patients. In short, a higher level of mCI in HD patients predicts longer survival in them.

### 4.3. Sarcopenia in HD patients

Sarcopenia is a condition in which there is a progressive loss of skeletal muscle mass and function with age [38]. In HD patients, PWE is prevalent due to chronic metabolic dysregulation, oxidative stress, chronic inflammation, and malnutrition. Sarcopenia, in turn, is one of main manifestations of PWE and thus tends to occur at a higher rate in the HD population. According to statistics, the prevalence of sarcopenia in HD patients has reached 28.5% [39].

As a result of sarcopenia, HD patients often manifest a significant decrease in muscle strength and physical function, which greatly increases the risk of falls and fractures in them [40]. At the same time, patients will consciously reduce their physical activities due to fear brought about by falls, which in turn leads to further development of sarcopenia and ultimately a vicious circle [41]. In addition, there is an increase in risk for cardiovascular events and death as sarcopenia affects the quality of life and physical function in patients [42]. There is still no consensus on the diagnosis of sarcopenia. There are independent expert consensuses in various regions, e.g., those proposed by the Asian Working Group for Sarcopenia (AWGS), Foundation for the National Institutes of Health (FNIH), and European Working Group on Sarcopenia in Older People (EWGSOP). In Asia, ‘possible sarcopenia’ has been developed as an additional diagnostic criterion [43]. These cumbersome diagnostic criteria not only require specially trained healthcare professionals, but also take up a significant amount of medical time to complete a series of tests they require, and as a result, only less than 25% of physicians report having used some of such criteria to diagnose sarcopenia [44]. In contrast, mCI only requires the collection of some routine data from HD patients, together with a simple formula to accomplish diagnosis of sarcopenia [10]. We summarized three currently published references relevant to topic and performed a meta-analysis. The results showed that mCI had good diagnostic accuracy for sarcopenia in both male and female HD patients. Hence, it can be a potential tool for identifying sarcopenia in HD patients.

Currently, most patients treated with long-term HD choose to undergo it in secondary or local hospitals. However, there may be a discrepancy between them and large medical institutions with respect to the skills and expertise of healthcare professionals, the sophistication of medical equipment, and the level of management in the hospitals. This discrepancy may result in failure to detect change in HD patients’ condition in a timely manner, thereby delaying treatment and increasing the risk of poor prognosis. Our study proposed an mCI derived from substituting patients’ conventional clinical indicators into a simple formula. The mCI can effectively predict the prognosis of HD patients, including nutritional status, risk of death, survival rate, and sarcopenia. The mCI is simple to calculate and easy to apply in clinical practice, providing clinicians with a powerful tool to more accurately assess and manage HD patients. In addition, to effectively integrate the mCI into clinical practice, we recommended that ① relevant healthcare professionals be trained regarding the mCI as a means of ensuring that they understand the importance and use of the mCI; ② mCI be included as part of the routine care of HD patients, and the mCI be monitored and recorded on a regular basis; and ③ the treatment plan be adjusted and the therapeutic effects be monitored according to the mCI.

Limitations: ① This study analyzed multiple predefined outcome measures separately by including 18 studies. However, not all outcome measures are well supported by the literature, although its broad information provides a study with comprehensive data support. For outcome measures included in a limited amount of literature, the results of analysis of them are less reliable. ② In this study, most of the included studies were from Asia, and this geographic bias may pose a limitation on the generalizability of our findings. ③ Some of the meta-analysis results in a study were highly heterogeneous. This may pose a challenge to the consistency and reliability of the results. Although this study used a random-effects model and conducted sensitivity analysis, meta-regressions and subgroup analysis to find out the sources of heterogeneity, these efforts did not fully address the heterogeneity problem. ④ This study was unable to adequately discuss possible confounders, which may not have been adequately identified or controlled during analysis. Future studies should consider these potential confounders in greater depth and adjust for these key variables in analysis to enhance the precision of findings.

## 5. Conclusions

The results of this study suggest that mCI can be used as a prognostic marker for HD patients. However, the number of the included studies is still limited, and the generalizability and accuracy of their results still need to be further validated and confirmed by more high-quality studies. It is also hoped that future studies will not only focus on the prognostic role of the mCI in HD patients, but also track the interventional outcomes of the mCI in clinical application.

## Supplementary Material

Supplemental Material

## Data Availability

All data generated or analyzed during this study are included in this published article.
