# Supplementary material for: Modified creatinine index for predicting prognosis in hemodialysis patients: a systematic review and meta-analysis
Source: Ren Fail. 2024 Aug 9;46(2):2367026. doi: 10.1080/0886022X.2024.2367026 (PMC11318488; doi:10.1080/0886022X.2024.2367026)
Supplement: Supplemental Material [file IRNF_A_2367026_SM8162.docx]

Supplementary Materials


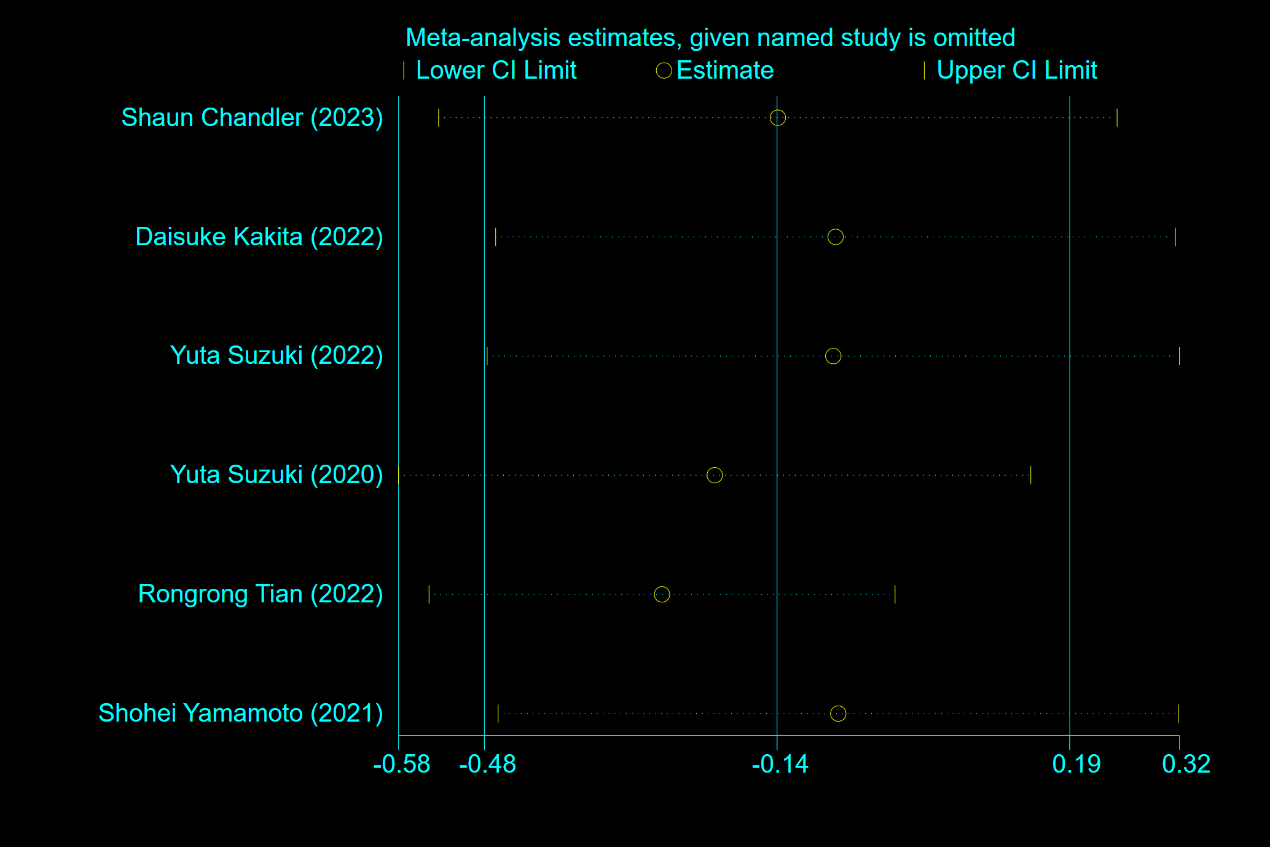


Figure 1. Sensitivity analysis of the association between low and high levels of mCI and the duration of maintenance of dialysis in HD patients


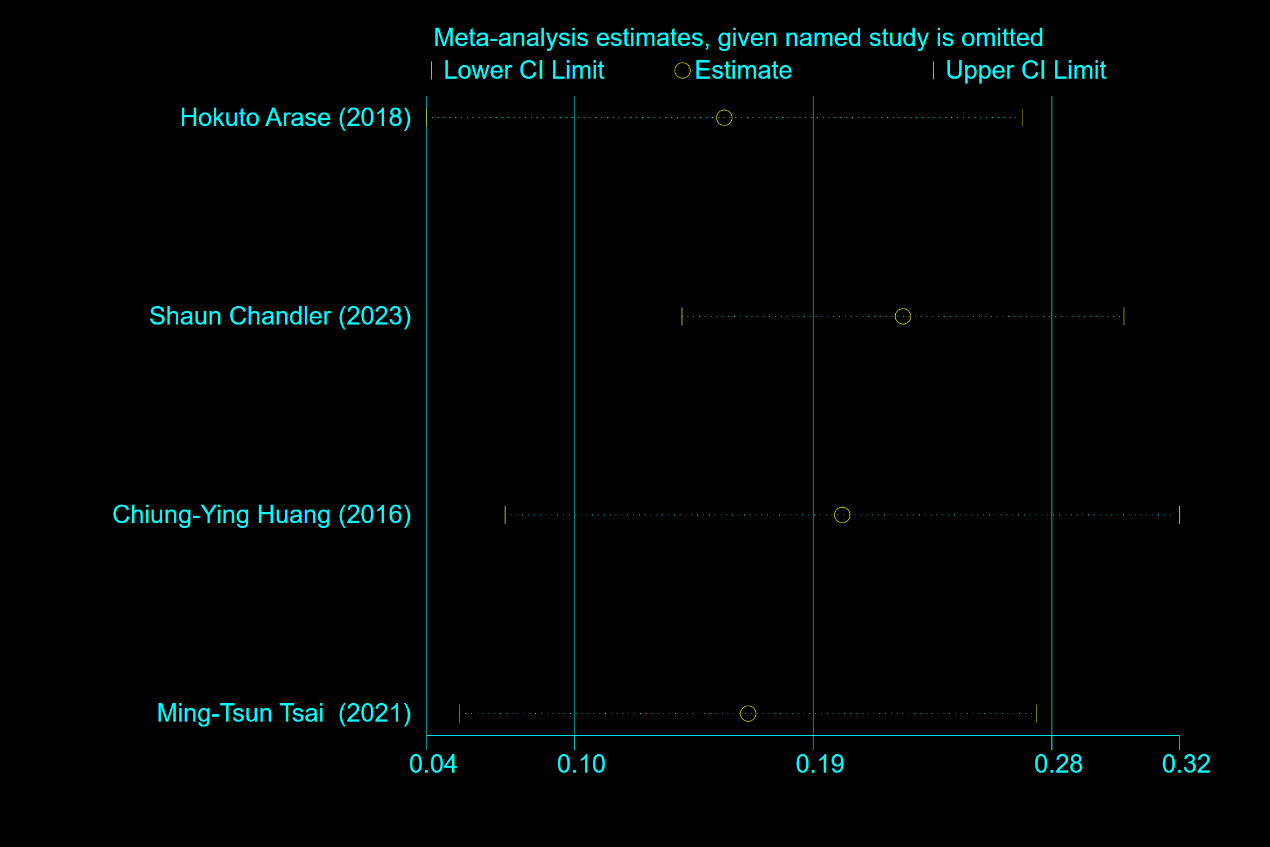


Figure 2. Sensitivity analysis of the association between mCI and BMI in HD patients


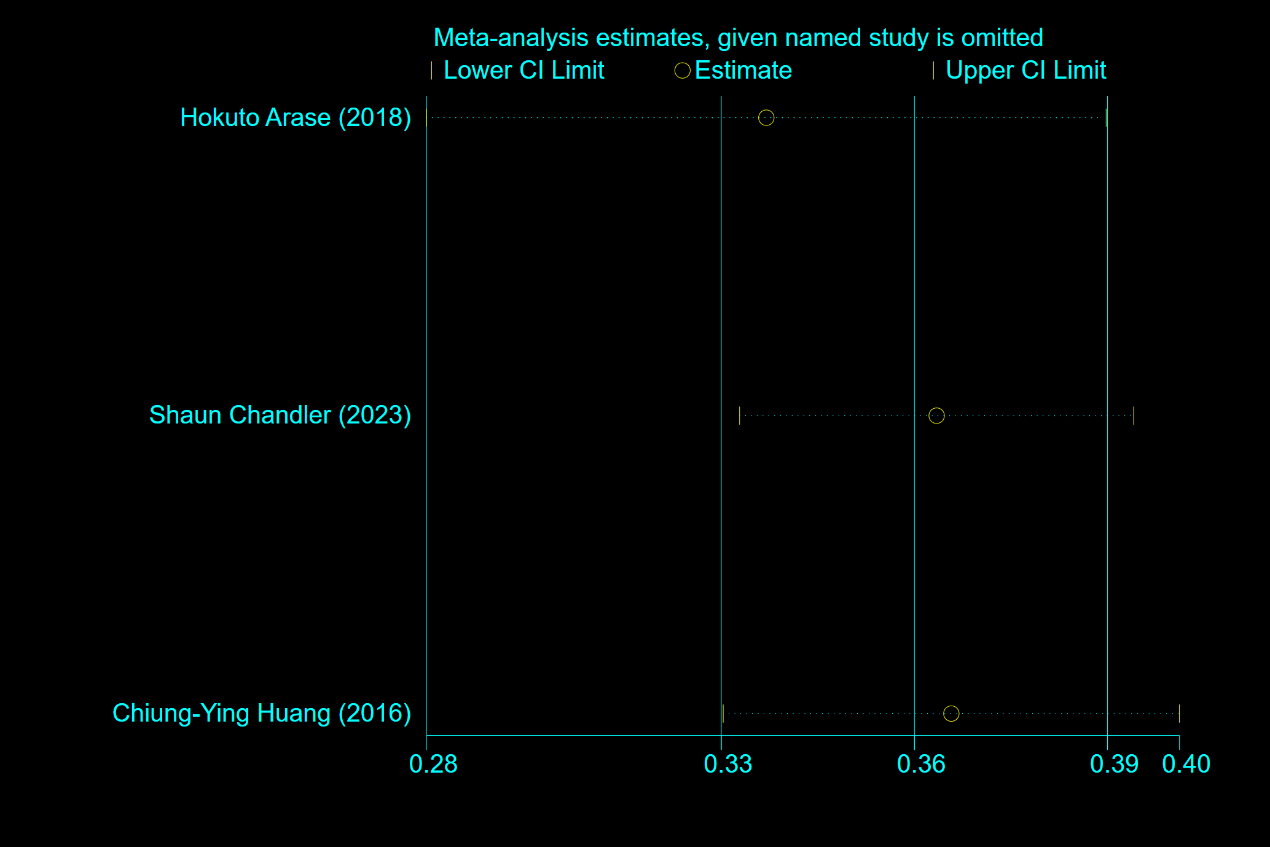


Figure 3. Sensitivity analysis of the association between mCI and albumin in HD patients


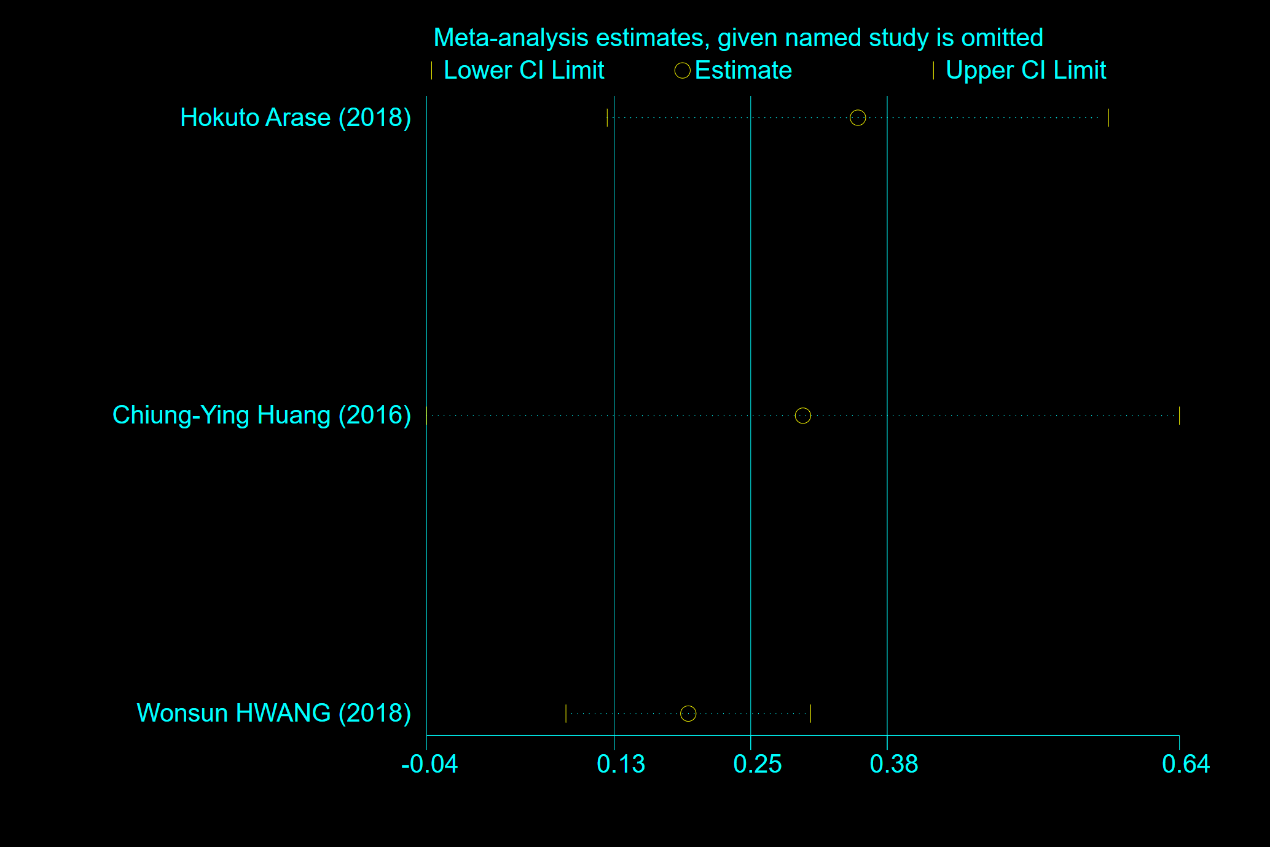


Figure 4. Sensitivity analysis of the association between mCI and nPCR


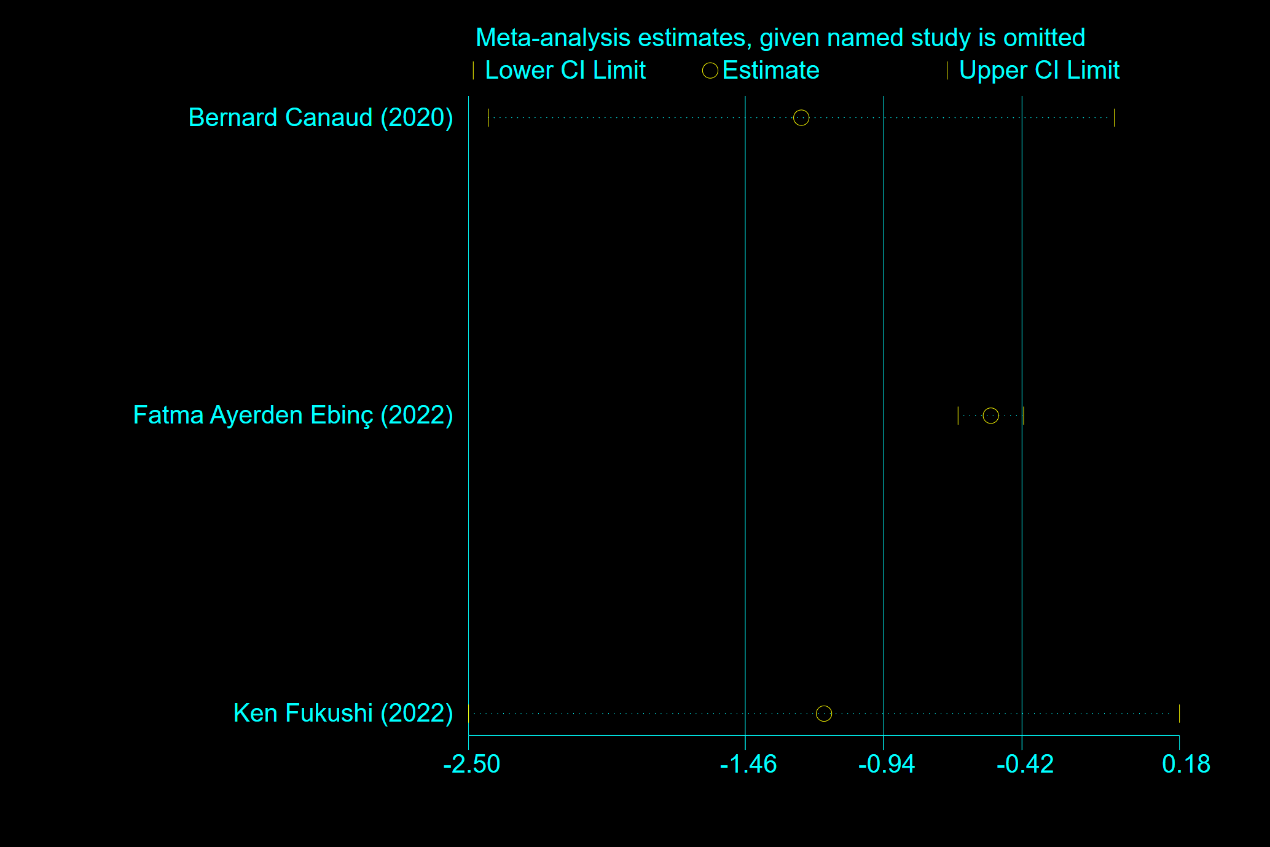


Figure 5. Sensitivity analysis of the association between mCI and survival/death in HD patients


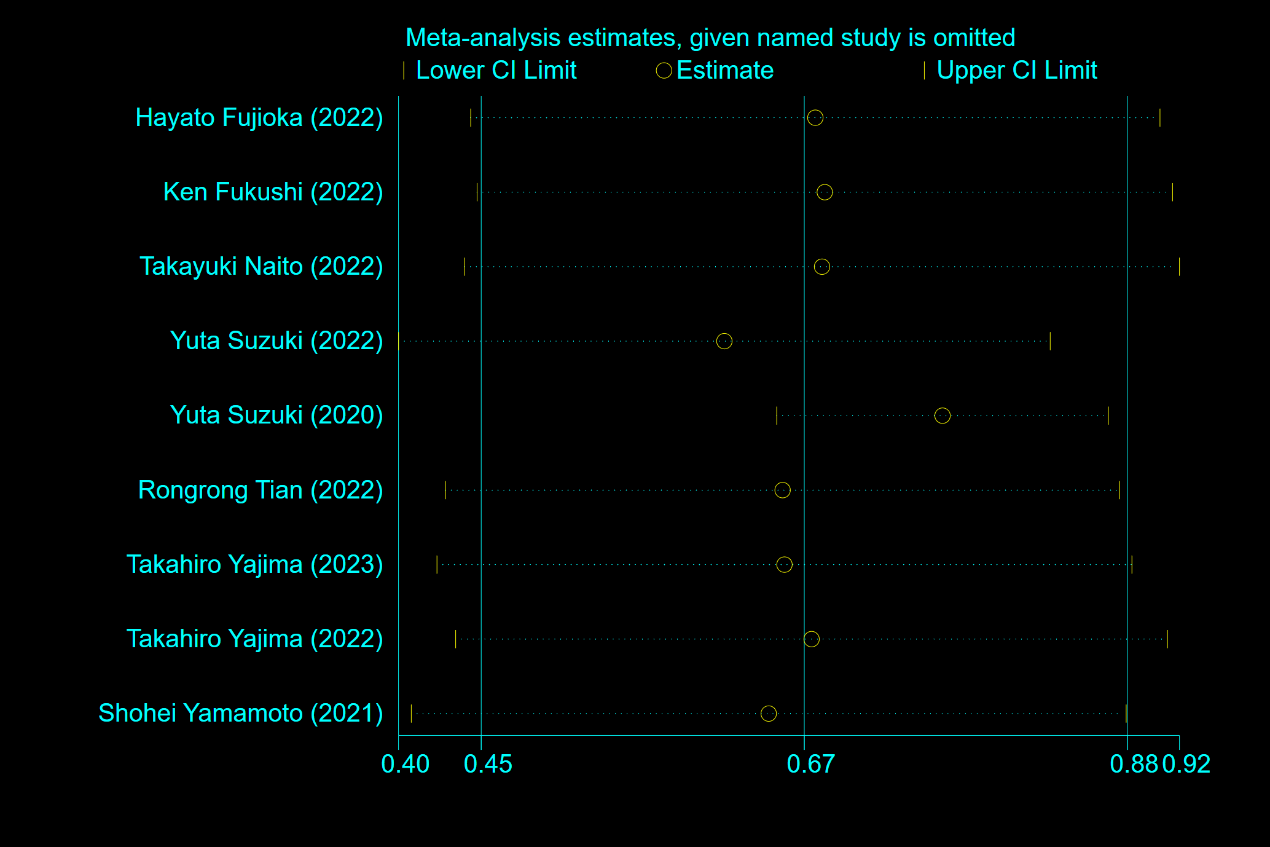


Figure 6. Sensitivity analysis of the association between mCI and risk of all-cause death in HD patients


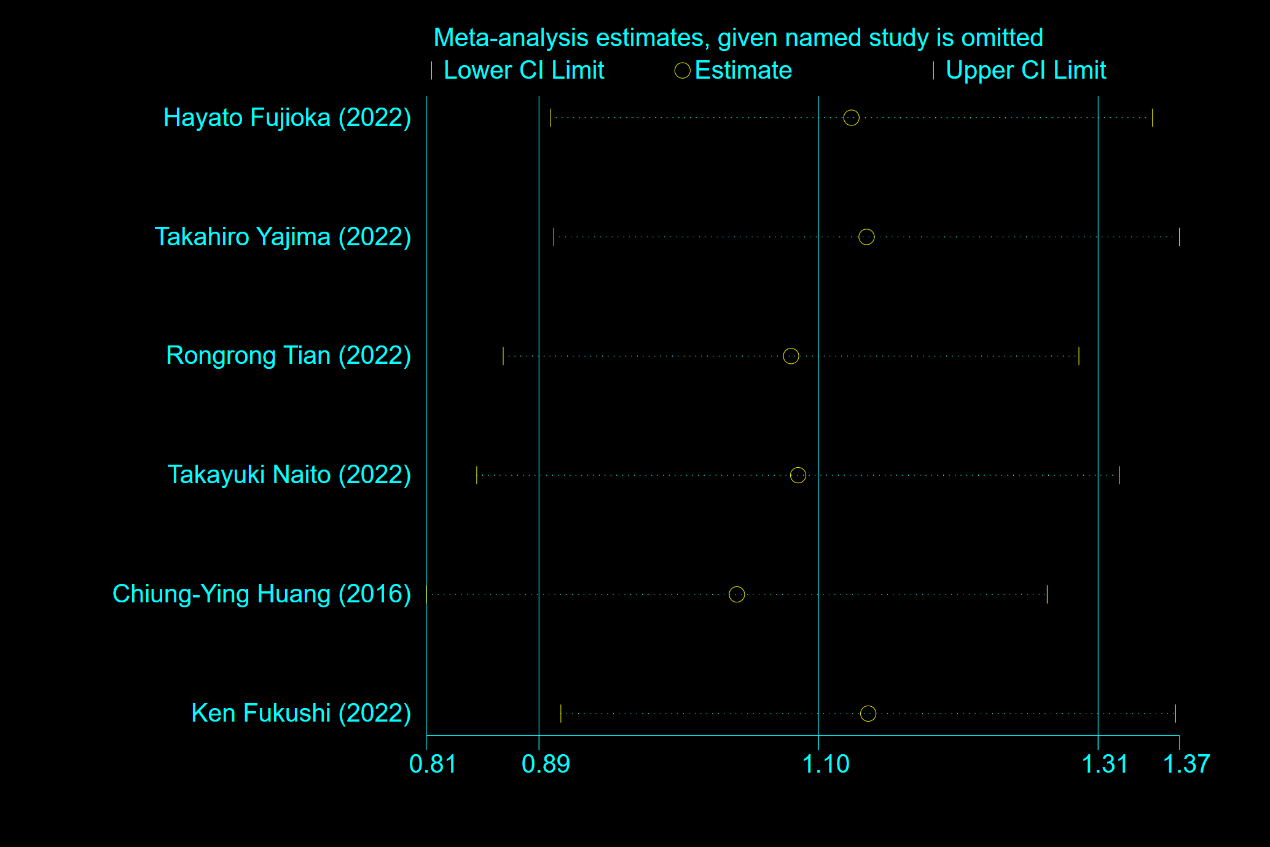


Figure 7. Sensitivity analysis of the association between mCI and OS in HD patients
